# Does perceptual grouping improve visuospatial working memory? Optimized processing or encoding bias

**DOI:** 10.1007/s00426-021-01555-w

**Published:** 2021-07-08

**Authors:** Antonio Prieto, Vanesa Peinado, Julia Mayas

**Affiliations:** 1 Department of Basic Psychology I, Universidad Nacional de Educación a Distancia, C/Juan del Rosal, 10, 28040 Madrid, Madrid Spain; 2 Department of Personality, Evaluation and Clinical Psychology, Universidad Complutense de Madrid, Campus Somosaguas, 28223 Pozuelo de Alarcón , Madrid Spain; 3 Department of Basic Psychology II, Universidad Nacional de Educación a Distancia, C/Juan del Rosal, 10, 28040 Madrid, Madrid Spain

## Abstract

Visual working memory has been defined as a system of limited capacity that enables the maintenance and manipulation of visual information. However, some perceptual features like Gestalt grouping could improve visual working memory effectiveness. In two different experiments, we aimed to explore how the presence of elements grouped by color similarity affects the change detection performance of both, grouped and non-grouped items. We combined a change detection task with a retrocue paradigm in which a six item array had to be remembered. An always valid, variable-delay retrocue appeared in some trials during the retention interval, either after 100 ms (iconic-trace period) or 1400 ms (working memory period), signaling the location of the probe. The results indicated that similarity grouping biased the information entered into the visual working memory, improving change detection accuracy only for previously grouped probes, but hindering change detection for non-grouped probes in certain conditions (Exp. 1). However, this bottom-up automatic encoding bias was overridden when participants were explicitly instructed to ignore grouped items as they were irrelevant for the task (Exp. 2).

## Introduction

Visual working memory (VWM) is a temporary buffer capable of actively storing, processing and manipulating the information coming from the visual world (Logie, 1986; Luck & Vogel, 2013). However, VWM is severely restricted in terms of capacity, with estimates converging on a limit of 3–4 items stored simultaneously (e.g., Alvarez & Cavanagh, 2004; Awh et al., 2007; Fukuda et al., 2010; Luck & Vogel, 1997). These capacity constraints lead to two different questions: First, can these storage limits be exceeded? And second, if so, what factors may contribute to increasing the capacity of VWM?

Behavioral, neurophysiological and even daily-life evidence has shown that VWM capacity limits can be surpassed. Moreover, several cognitive (e.g., long-term memory aids, familiarity, attentional selection) and perceptual factors seem to affect VWM capacity (Brady et al., 2016; Heuer & Schubö, 2016; Jackson & Raymond, 2008; Qian et al., 2017). Among these perceptual factors, organizational cues and particularly the Gestalt grouping principles (e.g., similarity, proximity, closure, common fate, continuity) have been a prolific area of interest (Palmer, 2003; Pomerantz & Kubovy, 1981; Wagemans, 2016). These organizational cues are known to improve perceptual performance (see Wagemans et al., 2012 for an extensive review; Wertheimer, 1923) by parsing the visual scene into component objects according to certain rules (Duncan, 1984; Duncan & Humphreys, 1989; Kahneman & Henik, 1977; Moore & Egeth, 1997). Particularly, perceptual grouping seems to parse the visual scene at a preattentive stage of visual processing and organizes the visual information before other cognitive processes have access to it (Lamy et al., 2006; Mack et al., 1992). Nonetheless, even if grouping occurs pre or even inattentively, attention is needed for the items to access and be encoded into working memory (Moore & Egeth, 1997). Hence, it would be not surprising if this early organization of the visual information could improve information processing (Woodman et al., 2003) leading to an increase in VWM performance, but the critical question is whether this early organization of the visual information also unintentionally biases the allocation of attentional resources over the items to be memorized (Duncan, 1984; Qian et al., 2020).

There is consistent evidence of improved VWM performance when the items to be remembered can be linked by the presence of different grouping cues (e.g., Allon et al., 2019; Gao et al., 2016; Peterson et al., 2015; Peterson & Berryhill, 2013; Woodman et al., 2003; Xu & Chun, 2007; Zhang et al., 2016; but see Li et al., 2018 for a review of grouping effects on VWM). For example, Woodman et al. (2003), using a pre-cued change detection task (CDT), found that grouping the items through spatial proximity improved VWM performance. Their results showed that grouped items tend to be stored together even if they are not directly cued. The grouping principle of similarity has also been widely studied in the context of VWM benefits. In fact, two different studies, Peterson and Berryhill (2013) and Peterson et al. (2015) found that color similarity increased VWM performance in a classical CDT. Other grouping principles like closure, connectedness and collinearity have also been proven to be effective in improving the performance of VWM, but the effects sizes obtained are significantly lower than those obtained in studies that employ the grouping principles of proximity and similarity (Gao et al., 2016).

Although there is compelling evidence on the effects of perceptual grouping over VWM performance, the mechanisms behind this effect are less clear. One possibility is that perceptual grouping only serves as a mean to organize visual information, allowing multiple individual items that share common features (like some grouping principles) to be treated as a single unit in memory or a “chunk”, a form of lossless compression that leads to more efficient processing of the information (Brady et al., 2009; Corbett, 2017; Nassar et al., 2018; Zhang & Luck, 2011). This would allow more items to be stored by freeing up resources that can be reallocated to other items (e.g., Bays & Husain, 2008), or using fewer slots for the same amount of information. In agreement with this account of grouping effects, Kałamała et al. (2017) found that the total number of objects maintained in VWM (*k* value) was greater when some of the items to be remembered were grouped by whole-part similarity. Moreover, Peterson et al. (2015) found that benefits to VWM derived from different grouping principles (similarity, proximity and connectedness) were echoed by a reduction of contralateral delay activity (CDA) amplitude, a result that can be interpreted in terms of a reduction of the resources devoted to VWM. On the other hand, the improvement in VWM performance could be derived from an encoding bias towards the grouped items (Li et al., 2018; Peterson & Berryhill, 2013). According to this account, the VWM improvement would result from the preattentional processing of grouping cues and the object-based (or feature-based) attentional capture provoked by the grouped elements (Treisman, 1982; Vecera, 1994; Vecera & Farah, 1994). This will result in a better encoding of the grouped items into the VWM at the expense of a poor encoding of the non-grouped items, especially when the number of items exceeds the VWM capacity (Awh et al., 2006). In support of this explanation, Peterson and Berryhill (2013) found that the VWM was restricted to probed items that were grouped during stimulus presentation, suggesting a bias toward encoding the grouped items. Moreover, Qian et al. (2020) using a pre-cued CDT paradigm, showed that certain features (such as color) guide the attention in a mandatory and automatic manner, leading to a memory benefit for the items that shared this feature. Finally, in a recent meta-analytic study, Li et al. (2018) provided a tentative explanation for the mechanisms behind the effect of grouping on VWM. These authors argued that the grouping effect seems to depend on the grouping relevancy of the tested feature. Specifically, the authors suggest that when the tested feature is grouping relevant (as is the case of our study), the feature is processed first to form perceptual grouping, and its storage will not involve competing for attention or memory resources as grouping seems to occur at a preattentive stage (Duncan & Humphreys, 1989; Mack et al., 1992). However, if an irrelevant feature is tested, storage for that feature should occur after the perceptual group is perceived and the items will compete with the others to obtain better storage. In this case, irrelevant features will obtain attentional priority in the encoding stage due to their belonging to the grouped items (Fine & Minnery, 2009; Melcher & Piazza, 2011).

Given the mixed evidence found in the previous literature regarding the nature of the VWM improvements, the present study aims to explore the mechanisms that underlie the VWM improvement associated with perceptual grouping and, especially, the role that attentional processes play in it when grouping-relevant features are tested. We focused on two possible accounts for this beneficial effect. The first is a “chunking” process that compresses grouped items into a single “chunk” of information. This hypothesis states that grouping allows more efficient processing of the items to be remembered without compromising attentional resources, so more resources would be available to store the relevant information (Corbett, 2017). Conversely, the “encoding bias” hypothesis states that the presence of grouping cues is processed pre-attentively at the early stages of the perceptual stream, and automatically directs feature-based attention to the grouped elements (Duncan, 1984; Qian et al., 2020; Vecera, 1994) during the memory task. This last approach leads to an attentional encoding bias that would impair the encoding of the non-grouped items into the VWM. To this end, we combined a classical CDT and the use of a variable-delay retrocue paradigm to direct the attention to specific item locations within the internal representations of the items stored in the VWM.

To this end, we conducted two experiments in which an array of six colored items were presented briefly at six different locations. In Experiment 1, the participants were instructed to memorize all the items until the appearance of a probe, which could either have the same or different color (see Rensink, 2002 for a thorough review on change detection theoretical basis and methodologies). Two critical manipulations were added to the main task: (1) in some trials, two of the items of the memory array shared the same color (grouped by color similarity), and (2) three different endogenous retrocue conditions with variable delays were included. As previous literature indicates that the benefit of perceptual grouping occurs at the encoding stage of the VWM (Li et al., 2018), we expect different effects depending on the delay of the retrocue. In the short-retrocue condition, the retrocue appears while the iconic-memory trace of the array persists (Becker et al., 2000; Gegenfurtner & Sperling, 1993; Sperling, 1960). Thus, we expect similar facilitation in the change detection task for any cued item, regardless of whether it was or not previously grouped, as all the information of the memory array will still be available independently of the attentional resources allocated to each item. In the long-retrocue condition, the retrocue appears after the iconic memory trace has vanished and the array to be remembered is already encoded in the VWM. If grouping not only organizes the visual scene but also induces an attentional encoding bias towards grouped items, then we expect the retrocue to have different effects depending on the items cued. In this case, grouped items will be better encoded and less prone to decay over time as more attentional resources are allocated to them. This would result in the cue being more effective for the grouped items.

In Experiment 2, participants were instructed to ignore the items that shared the same color (grouped items), as they would not be probed in the CDT. This allows us to test whether an encoding bias towards grouped items can be counteracted by top-down voluntary processes that filter out irrelevant information according to the goals of the task or whether, on the contrary, grouped items can capture attention irrespective of the task demands. The rationale for this experiment was twofold. First, according to our encoding bias hypothesis, it would be interesting to address how the task demands could affect the capacity of the grouped items to bias the encoding of non-grouped items and, therefore, memory performance. Second, there is conflicting evidence in the previous literature regarding whether perceptual grouping enhances or hinders the inhibition of irrelevant information. On one side, Kimchi et al., (2007, 2016) found that perceptual grouping can capture attention irrespective of task demands, leading to full processing of irrelevant items and hindering the inhibition of non-relevant information. A similar result was obtained by Zupan and Watson (2020) who found that perceptual grouping reduced the number of distractors that can be inhibited. On the other hand, Allon et al. (2019) showed that grouping by spatial proximity and the presence of illusory objects (another form of perceptual organization) improved filtering performance when the grouped items acted as distractors. However, given that different grouping principles are thought to have different processing demands (Driver et al., 2001; Kimchi & Razpurker-Apfeld, 2004) and participants are forced to attend and process the grouped stimuli to filter them out (as grouping is the key feature that distinguishes relevant from irrelevant items), it is not clear whether the similarity cues employed in the present study will lead to a better or worse filtering performance.

To sum up, in Experiment 1, we expect to find: (1) a general improvement in change detection accuracy in trials in which part of the items of the memory array are grouped through color similarity. (2) According to the encoding bias hypothesis, we expect that this improvement will only occur when the items probed are previously grouped in the memory array, and no benefit or even a worsening effect will appear when the probes are non-grouped items of a grouped memory array. 3) We also hypothesize that the non-grouped items will be more affected by the delay of the retrocue, due to the worse encoding and the increased susceptibility to decay over time. In Experiment 2 we hypothesize that, according to previous results in space-based presentations (Allon et al., 2019), participants will be able to counteract the attentional capture generated by grouped items and filter them out as they are irrelevant for the task. This will lead to an opposite pattern of results compared to that of Experiment 1, with better performance in change detection in those arrays that contain grouped elements (4 relevant elements) compared with arrays that do not contain grouped elements (6 relevant elements).

## Experiment 1

In Experiment 1, we aimed to investigate the effect of an always-valid retrocue presented at different latencies over the recognition of the elements of a memory array that could be all of different color or contain two adjacent items grouped by color similarity. We employed a change detection task (Pashler, 1988) in which participants were asked to indicate if the element presented as a probe was the same or different color as the element that appeared at the same position in the memory array.

### Methods

#### Participants

Sixty-two undergraduate students (fourteen males; age range: 18–51, mean age = 28.6; SD = 9.5) at the Universidad Nacional de Educación a Distancia (UNED) participated in this experiment as part of a practical course. Fifty-seven were right handed and all of them had either normal or corrected-to-normal visual acuity and color vision. The results of the a priori power analysis indicated that a minimum sample size of 54 participants was necessary to achieve a power of 0.80, given an effect size of *f* = 0.20 (medium). The study was conducted following the Declaration of Helsinki and it was approved by the Universidad Nacional de Educación a Distancia Ethics Committee. Written informed consent was obtained from the participants before starting the course.

#### Stimuli and apparatus

The stimuli consisted of six colored circles, subtending 2.8º of visual angle (VA), each made with Microsoft Paint software. The stimuli were arranged in a circular configuration (8.5º VA from the center of the screen to the center of each circle). Six color categories were used: red (*R*: 255; *G*: 0; *B*: 0), yellow (*R*: 255; *G*: 255; *B*: 0), green (*R*: 0; *G*: 255; *B*: 0), blue (*R*: 0; *G*: 0; *B*: 255), cyan (*R*: 0; *G*: 255; *B*: 255) and magenta (*R*: 255; *G*: 0; *B*: 255). These colors were combined to form two different types of arrays: (1) grouped arrays containing two adjacent circles that share the same color and four circles of different colors; and (2) non-grouped arrays in which all six circles had a different color. The color distribution and the position of the pairs of grouped stimuli in the arrays were randomly selected. The retrocue consisted of an arrow subtending 1.4º VA whose base was located at the center of the screen. The probe was a single-colored circle of the same dimensions as the ones presented previously and placed randomly in one of the six possible locations of the array. The experiment was programmed using E-Prime2 (Psychology Software Tools) and displayed on an LCD-IPS 24-inch widescreen monitor with a refresh rate of 75 Hz and a resolution of 1280 × 720 pixels. The viewing distance was kept constant at 60 cm.

#### Design and procedure

**Fig. 1:**
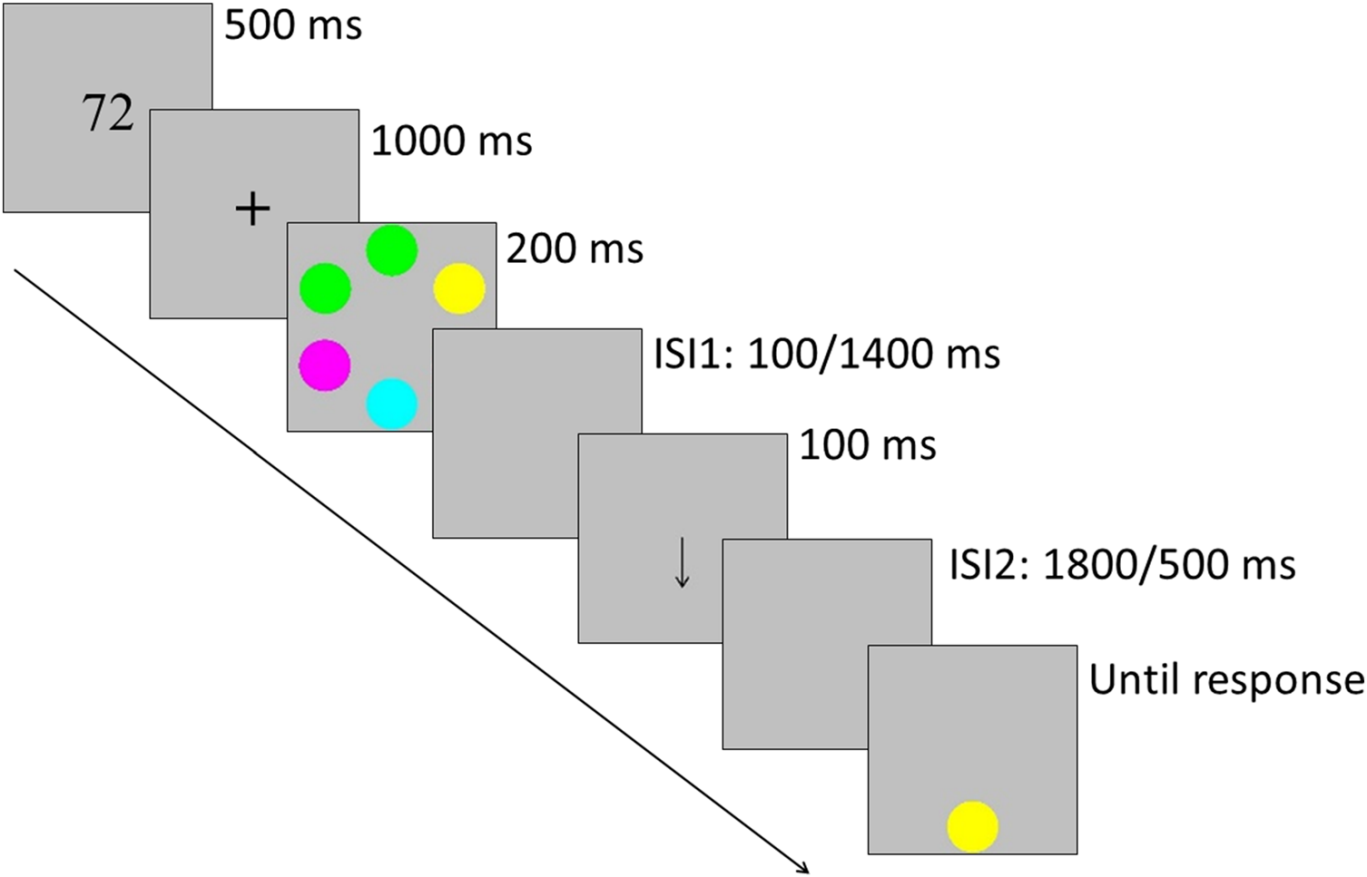
Experimental sequence in Experiments 1 and 2

A repeated-measures design with the within-subject factors: *retrocue latency* (short, long, no cue), *type of array* (grouped array[GA], non-grouped array [NGA]) and *probe grouping* (grouped [GP], non-grouped [NGP]) was employed. The design was not completely factorial, as the grouped probes were only possible within the context of arrays that contained grouped elements. The task consisted of a variant of the classic change detection paradigm in which an always valid variable-delay retrocue, indicated the item to be remembered in some of the trials (see Fig. 1).

Participants were tested in individual booths in our laboratory. The task consisted of indicating, as fast and accurately as possible, whether the color of the probe matched the one positioned at the same location in the memory array (50% chance). Each trial began with the appearance of two digits (500 ms) that the participants must repeat aloud throughout the trial to avoid the verbal encoding of the colors and spatial locations that compounded the stimulus array (Matsukura & Vecera, 2015). After the offset of the two digits, a fixation cross appeared in the center of the screen (1000 ms) to ensure the central fixation of the eye gaze followed by the memory array (200 ms). After the disappearance of the memory array, an arrow indicating the position of the item that will be probed (retrocue) could appear at two different delays: in the *short-latency* condition, the retrocue was presented 100 ms after the memory array (ISI 1) and disappeared 1800 ms before the onset of the probe (ISI 2); in the *long-latency* condition, the retrocue appeared 1400 ms after the memory array and disappeared 500 ms before the onset of the probe. Finally, in the *no-retrocue* condition, the same gray background was presented until the appearance of the probe. In all retrocue latency conditions, the retrocue remained on the computer screen for 100 ms (or only the gray background in the no-retrocue condition), so that in all trials the time between the offset of the memory array and the onset of the probe was kept constant at 2000 ms. Finally, the probe was presented until the participant gave a response. Each retrocue latency condition represented 1/3 of the total trials. Participants were instructed to answer, as accurately and fast as possible, whether the color of the probe matched the one at the same location in the memory array (forced-choice response). The participants responded by pressing the “1” and “2” keys of the numeric keyboard. Half of the trials were “same” trials in which the probe had the same color as the item at the same location in the memory array, the other half were “different” trials, in which the probe color was different from the item at the same location in the memory array. Before the start of the experiment, participants performed an 8-trial practice block in which participants received feedback on their performance. The experiment consisted of 360 trials divided into two blocks of 180 trials with a short break between them. The total number of trials per condition was the same for all conditions and set to 40 (20 per block). All the experimental conditions were randomly presented, and response keys were counterbalanced across participants. No feedback regarding the accuracy of the responses was given during experimental trials.

### Results

Trials with reaction times (RTs) below 200 ms and those above/below 2.5 standard deviations from the mean were rejected before entering the accuracy analysis. In the RT analysis, trials with RTs above 2500 ms were also rejected. Statistical analyses were performed using repeated measures ANOVAs and post hoc comparisons when necessary (Bonferroni corrected). RTs and accuracy (% correct) were taken as dependent variables (Cowan, 2010; Peterson & Berryhill, 2013). The design of the experiment was not completely factorial as probes in non-grouped arrays (NGA) could only be non-grouped, so we performed a 3 × 3 repeated-measures ANOVA with the within-subjects factors *retrocue latency* (short, long, no retrocue) and *probe type* (NGA, GA/GP; GA/NGP).

#### Accuracy (% correct)

**Fig. 2:**
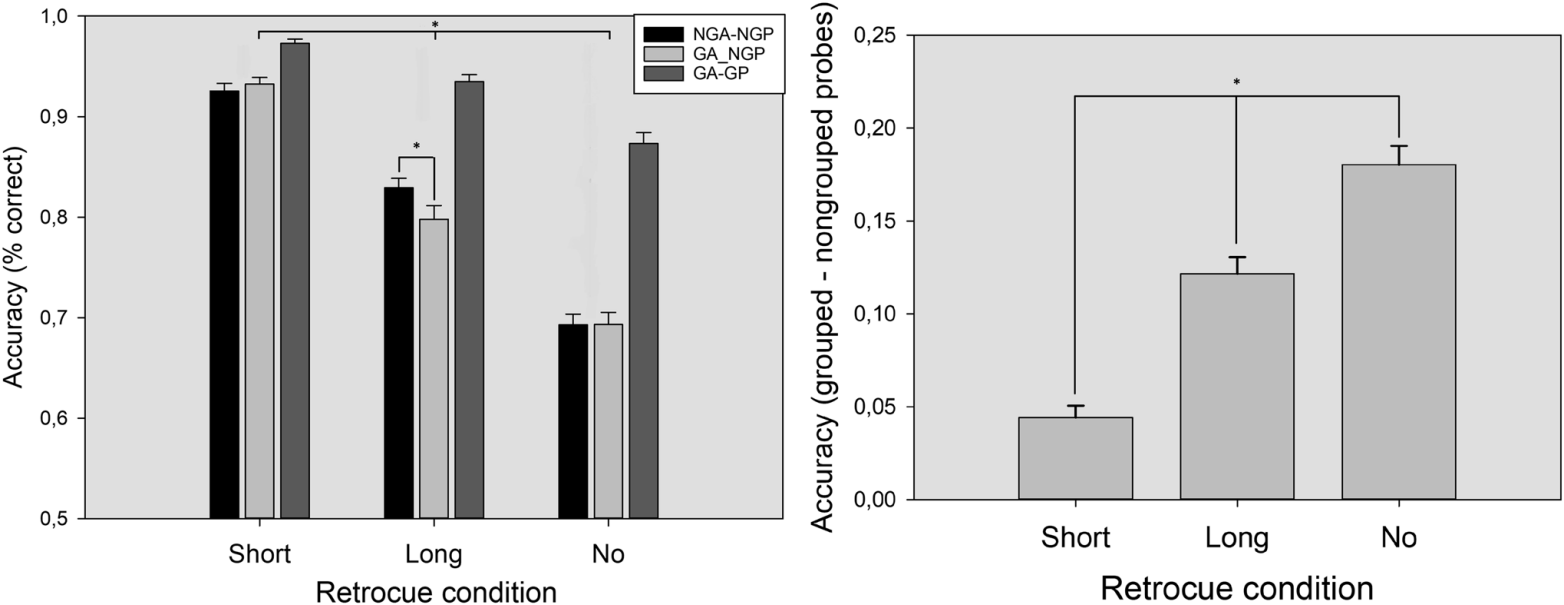
Accuracy for GP and NGP in both NGA and GA as a function of retrocue condition (**a**). Accuracy differences between grouped and non-grouped probes as a function of retrocue condition. Accuracy (% correct) for GP and NGP (left), and differences in accuracy for GP and NGP (right) in the three retrocue conditions (**b**). NGP: non-grouped probes, GP: grouped probes. The error bars represent 1 SEM

The results of the ANOVA showed a significant main effect of the factor *probe type* *F* (2,60) = 264.07; *p* < 0.001; *ŋ2p* = 0.812; *β* = 1. Post hoc pairwise comparisons showed that GP were better recognized (92.7%; all ps < 0.01) than NGP, whether they came from NGA arrays or from GA arrays (81.6% vs 80.8%, respectively; *p* = 0.26). The main effect of *retrocue latency* also reached statistical significance *F* (2,60) = 328.52; *p* < 0.001; *ŋ2p* = 0.843; *β* = 1. Pairwise comparisons indicated that the performance in the change detection task was maximal after short-retrocue trials (94.3%), followed by long-retrocue trials (85.4%) and no-retrocue trials (75.3%), respectively (all *p’s* < 0.001). Finally, these effects were qualified by a significant *retrocue latency x probe type* interaction *F* (4,58) = 33.26; *p* < 0.001; *ŋ2p* = 0.353; *β* = 1. Post hoc comparisons showed that NGP in NGA were better recognized compared with NGP in GA but only in the long-retrocue condition (82.9% vs 79.8%; *p* = 0.014). In addition, recognition performance for GP was better compared to NGP in all retrocue conditions (all *p*s < 0.001). Afterwards,, to analyze the effect of the different retrocue conditions over grouped and non-grouped probes, we computed a differential variable by subtracting accuracy for NGP from accuracy for GP (in both GA and NGA) in each retrocue condition [Accuracy GP – Accuracy NGP]. The one-way ANOVA performed on this variable showed a main effect of *retrocue condition F* (2,60) = 78.51; p < 0.001; *ŋ2p* = 0.563; *β* = 1. Pairwise multiple comparisons indicated that the recognition advantage for GP increased as a function of retrocue latency (all *p*s < 0.001), with the short latency leading to the smallest difference (4.4%), followed by the long-latency condition (12.1%) and no-retrocue condition (18.0%). An overview of the results is shown in Fig. 2.

**Fig. 3:**
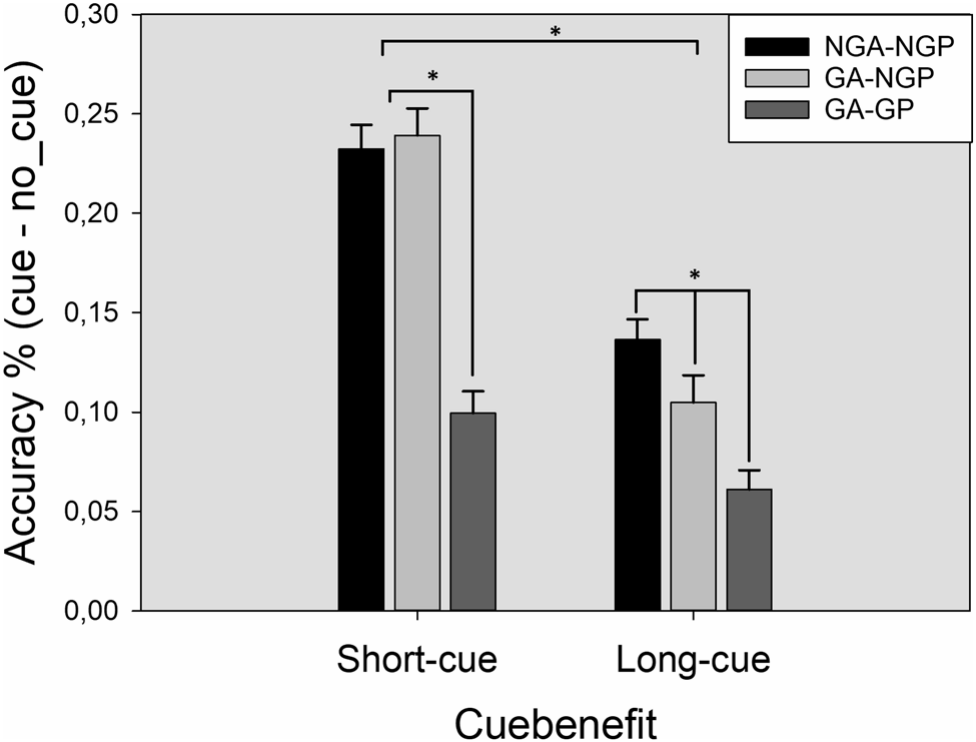
Cueing benefits in performance as a function of probe type.Benefits (accuracy % correct) of cueing (short and long retrocues) for NGA–NGP, GA–NGP and GA–GP (left). The error bars represent 1 SEM

Finally, to analyze the cueing benefits of short and long retrocues in each probe type (NGP, GP), we performed an additional ANOVA in Experiment 1 by computing a new differential variable that accounts for cueing benefits (the performance difference between valid and no retrocue conditions) for GP and NGP separately. This was carried out by subtracting the recognition performance in no-retrocue condition from the short- and long-retrocue conditions, respectively. Then, we performed a 3-*probe type* (NGP–NGA, NGP–GA, GP–GA) × 2-*cueing benefit* (short–no, long–no) repeated-measures ANOVA. The results showed a main effect of the factor cueing benefit *F* (1,61) = 181.73; *p* < 0.001; *ŋ2p* = 0.749; *β* = 1, indicating that the cueing benefit was higher with short delays of the retrocue (19% vs 10%). The main effect of probe type was also significant *F* (2,60) = 32.82; *p* < 0.001; *ŋ2p* = 0.350; *β* = 1. Pairwise comparisons showed that cueing benefits for NGP were larger than for GP (*p* < 0.001). Interestingly, the first-order interaction between cueing benefit and probe type also reached significance *F* (2,60) = 34.09; *p* < 0.001; *ŋ2p* = 0.358; *β* = 1. A closer look at this interaction revealed that while short retrocues are equally effective for NGP in both NGA and GA, long retrocues are more effective for NGP when the array did not contain grouped items (NGA), compared to arrays that contained grouped items (GA) *p* = 0.004. The results of these analyses are summarized in Fig. 3.

#### Reaction times

The analysis of the response times followed the same structure depicted in the accuracy analysis. We performed a 3 × 3 repeated-measures ANOVA, with *retrocue latency* (short, long, no retrocue) and *probe type* (NGA, GA/GP; GA/NGP) as within-subjects factors. The results showed a significant main effect of *probe type F* (2,60) = 103.48; *p* < 0.001; *ŋ2p* = 0.629; *β* = 1. Pairwise comparisons indicated that the responses for GP (746 ms) were faster than the responses for NGP (all ps < 0.001) regardless of whether they came from arrays with grouped items or not (833 ms and 821 ms, respectively). The main effect of *retrocue latency* was also significant *F* (2,60) = 630.46; *p* < 0.001; *ŋ2p* = 0.912; *β* = 1. Pairwise comparisons indicated that the responses in the change detection task were faster after short-retrocue trials (640 ms), followed by long-retrocue trials (745 ms) and no-retrocue trials (1016 ms), with all *p*’s < 0.001. Finally, the *retrocue latency x probe-type* interaction also reached statistical significance *F* (4,58) = 8.32; *p* < 0.001; *ŋ2p* = 0.120; *β* = 0.997. Post hoc comparisons revealed that the RTs for NGP in NGA were lower than RTs for NGP in GA, but only in the *no-retrocue* condition (*p* = 0.004). No other effects reached statistical significance. A summary of the RT results is shown in Table 1.

**Table 1:** Experiment 1 mean response times (SD) as a function of the type of array, probe grouping and retrocue latency

	Probe	Array type		
		GA		NGA
		NGP	GP	NGP
Retrocue latency	Short	652 (153)	637 (199)	654 (155)
	Long	781 (190)	699 (202)	769 (180)
	No	1056 (215)	989 (241)	1028 (204)

*GA* grouped array, *NGA* non-grouped array, *NGP* non-grouped probe, *GP* grouped probe. All response times are expressed in ms

### Interim discussion

The results of the first experiment indicated that Gestalt grouping by color similarity generally improves accuracy and RTs in a change detection task, a result that agrees with the results obtained previously in the literature (see Li et al., 2018). However, this effect seems to differ significantly between retrocue conditions and grouped/non-grouped probes. First, participants performed much better in the CDT when the probed items were previously grouped in the memory array, suggesting that the VWM benefit associated with grouping is restricted to those items. Second, the effect of the retrocue conditions was different between GP and NGP (see Fig. 2b). In short-retrocue trials, although performance for GP was higher, NGP almost matched them. However, the difference in performance between GP and NGP became larger as the retrocue increased in latency or if it did not even appear. Lastly, performance for NGP was not improved by the presence of grouped items. Instead, in the long-retrocue condition, performance for NGP worsened in GA compared to NGA trials and remained equal in the rest of the conditions. The RTs analysis mirrored this pattern of results, showing faster RTs in those conditions where participants were more accurate (see Table 1).

To sum up, three different findings support the encoding bias hypothesis in Experiment 1. First, we found a general improvement in change detection accuracy when some of the items in the memory array were grouped, but this benefit was restricted to GP. Second, the advantage for GP became smaller as the retrocue reduced its latency. Third, the presence of GP in the memory array did not improve recognition performance for NGP (or even worsened it depending on the conditions).

## Experiment 2

Overall, the results from Experiment 1 suggest that the VWM improvement found when the memory array contained grouped items was not caused only by a chunking process or the optimized organization of the information (that reduces the number of effective elements to be encoded), but by the automatic attentional capture produced by the grouped elements which, in turn, can hinder the encoding of the remaining items in the VWM (Awh et al., 2006; Edward Awh & Jonides, 2001; Treisman, 1982).

In Experiment 2, we explored whether this grouping-directed encoding bias could be overridden by the voluntary allocation of attentional resources that simultaneously select task-relevant and filter out irrelevant information (Allon & Luria, 2017; Allon et al., 2019; Li et al., 2017; Sawaki & Luck, 2010, 2011). To this end, we employed the same task and experimental design described in Experiment 1, but this time the grouped elements served as distractors, and participants were explicitly instructed to ignore them as they would not be tested in the CDT.

### Methods

#### Participants

Forty undergraduate students (ten males; age range 18–57, mean age = 25.05; SD = 9.92) at the Universidad Nacional de Educación a Distancia (UNED) participated in this experiment as part of their practice credits. Thirty-seven were right handed and all of them had either normal or corrected-to-normal visual acuity and color vision. The results of the a priori power analysis indicated that a minimum sample size of 42 participants was necessary to achieve a power of 0.80 given an effect size of *f* = 0.20 (medium). Approval and informed consent followed the same protocols as Experiment 1.

#### Apparatus and stimuli

The same stimuli and apparatus were used as in Experiment 1.

#### Design and procedure

The experimental design for Experiment 2 was a full-factorial repeated-measures design with 2 within-subject factors: 3 × *retrocue latency* (short, long, non-cue) and 2 × *type of array* (GA, NA). The task and procedure were the same as in Experiment 1, except for the following: (1) the instructions explicitly emphasized that “if in the memory array two items that share the same color appear, you must ignore these items as you will never be asked for them”. (2) Given that grouped items acted as distractors, probe items were always non-grouped elements of the memory array. (3) Experiment 2 consisted of 240 trials (40 trials per experimental condition), divided into two blocks of 120 trials with a pause between them. As in Experiment 1, all the experimental conditions were randomly presented and response keys were counterbalanced across participants. No feedback regarding the accuracy of the responses was given during experimental trials.

### Results

The rejection criterion for invalid trials and the analysis strategy followed the same rules specified in Experiment 1. The design of Experiment 2 was completely factorial, so we performed a repeated-measures ANOVA on accuracy (% correct) scores and RTs that included both factors.

#### Accuracy (% correct)

A 3 × 2 repeated-measures ANOVA, with the within-subjects factors *retrocue latency* (short, long, no retrocue) and *array type* (GA, NGA) was performed on accuracy scores (% correct). The main effect of the *retrocue latency* reached statistical significance *F* (2,38) = 83.40; *p* < 0.001; *ŋ2p* = 0.681; *β* = 1. Multiple pairwise comparisons showed differences between all retrocue conditions. Participants reached their best performance in the short-retrocue condition (90.2%), followed by long- (79.6%) and no-retrocue (70.1%) conditions, respectively. The main effect of *array type* was also statistically significant *F* (1,39) = 25.69; *p* < 0.001; *ŋ2p* = 0.397; *β* = 0.99. Accuracy for GA (81.6%) was significantly better than accuracy for NGA (78.3%).

**Fig. 4:**
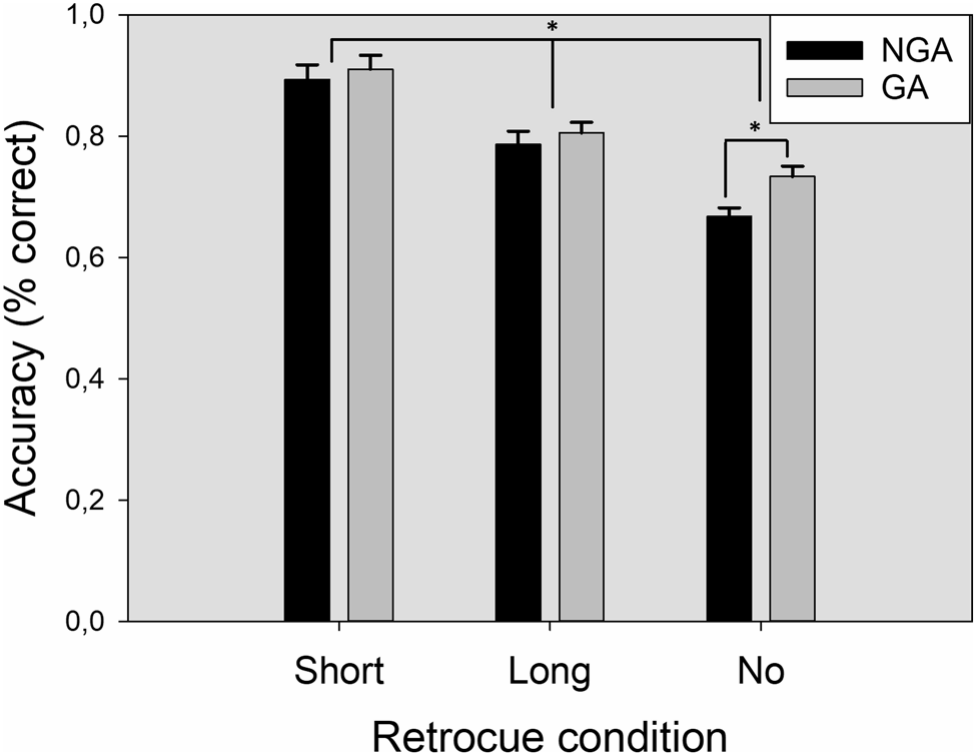
Accuracy as a function of retrocue condition and grouped items in the array. Accuracy (% correct) for arrays with and without grouped elements as a function of the three retrocue conditions. *NGA* non grouped arrays, *GA* grouped arrays. The error bars represent 1 SEM

Finally, the significant interaction effect between *retrocue latency* and *array type F* (2,38) = 4.20; *p* = 0.02; *ŋ2p* = 0.097; *β* = 0.722 qualifies the main effects reported. Post hoc comparisons revealed that the better performance for GA only reached statistical significance (*p* < 0.001) in no retrocue trials (73,4% vs 66.8%). No other effects reached statistical significance. A summary of the accuracy results is shown in Fig. 4.

#### Reaction times

RTs were analyzed through a 3 × 2 repeated-measures ANOVA, with the within-subjects factors *retrocue latency* (short, long, no-retrocue) and *array type* (GA, NGA). The main effect of the *retrocue latency* reached statistical significance *F* (2,38) = 148.94; *p* < 0.001; *ŋ2p* = 0.792; *β* = 1. Multiple pairwise comparisons carried out to break drown this effect showed that participants were faster in responding to the short-retrocue condition (742 ms), followed by long- (926 ms) and no-retrocue (1211 ms) conditions, respectively. No other main effects nor interactions were significant. A summary of the RTs for each condition can be seen in Table 2.

**Table 2:** Experiment 2 mean response times (SD) as a function of the type of array and retrocue latency

	Array type	
	NGA	GA
Retrocue latency		
Short	737 (292)	748 (302)
Long	908 (332)	945 (377)
No	1230 (552)	1193 (402)

*GA* grouped array, *NGA* non-grouped array. All response times are expressed in ms

### Interim discussion

Experiment 2 yielded an opposite pattern of results compared to Experiment 1 in both accuracy and RTs. The effect of the different retrocue conditions remained the same, but the critical change appeared when comparing performance between NGA and GA arrays. In Experiment 2, where participants were explicitly instructed to ignore grouped items, there was a clear benefit in terms of accuracy and RTs for GA, especially in the absence of a retrocue (as shown by the significant interaction between array type and retrocue condition). These results indicate that grouped items can be filtered out according to task goals, a result that agrees with those of Allon et al. (2019), who also found improved filtering performance in VWM when the irrelevant information was perceptually grouped. Thus, although perceptual grouping seems to have the capacity to automatically capture attention towards grouped items and bias the entry of information into VWM, it can also be inhibited following the task demands.

## General discussion

The purpose of the current study was to examine the effects of perceptual grouping (color similarity) over the VWM performance, and the mechanisms behind this performance improvement (e.g., Gao et al., 2016; Li et al., 2018; Peterson & Berryhill, 2013; Peterson et al., 2015; Woodman et al., 2003; Zhang et al., 2016).

The results from Experiment 1 support the encoding bias hypothesis, at least based on three convergent outcomes. First of all, although there is a general benefit in change detection when some items can be grouped in the memory array, a closer look at this effect shows that this benefit only appeared for GP and turned into a detrimental effect when NGP were tested (see Fig. 2 left). If the presence of grouping items only affects the memory task by allowing more efficient processing of the information to be stored without any effect on the attentional resources allocated to each item, then we should expect a similar benefit (and similar performance) for both grouped and non-grouped probes (Corbett, 2017; Nassar et al., 2018). Second, the effect of the retrocue conditions differed between GP and NGP. Particularly, these differences progressively increased as the cue presentation interval became longer (see Fig. 2 right). This effect could be explained by the weaker encoding of non-grouped items and the increased decay rate of the memory trace over time (Barrouillet & Camos, 2012) or, alternatively, by a lower probability of non-grouped items to be encoded due to the lower attentional resources allocated to them (Anderson et al., 2013; Zhang & Luck, 2008). If the grouping effect only affects the organization of the information without biasing attentional, we should expect a similar effect of the different retrocue latencies for all the items in the memory array. Third, the presence of grouped items in the memory array (GA trials) did not improve change detection performance for NGP compared to NGA trials (see Fig. 2 left) as would be expected if perceptual grouping only organized the visual information without biasing the encoding of the items to be stored. Particularly, based on a pure “chunking” hypothesis, grouped items should be treated as a single object (reducing the number of effective items to be memorized to five) and, therefore, we would expect a better overall performance in trials containing grouped items (GA) compared to trials without grouped items (NGA). Instead, we found a mixed pattern of results depending on the retrocue condition. In long-retrocue trials, the presence of grouped elements in the memory array worsened change detection performance for NGP compared to arrays without grouped elements, a decrease in performance that did not appear in short-retrocue trials (see Fig. 2 left and Fig. 3). This pattern of results could be explained by a worse encoding of non-grouped items when other grouped items are present. In short-retrocue trials, even if the non-grouped items are poorly encoded due to the presence of grouped items, the aid of the retrocue presented when the iconic memory trace is still available helps to maintain the performance level. However, in long-retrocue trials, the poorer encoding and higher sensitivity to decay over time of the non-grouped elements caused by the presence of grouped items lead to a performance drop compared to trials in which no grouped elements appear. Surprisingly, this drop in performance did not appear in the no-retrocue condition, a result that we expected to find according to the encoding bias hypothesis (even to a greater extent than in long-retrocue trials). One possible account for this absence of a significant effect in the no-retrocue condition could be a floor effect in change detection performance. This explanation is supported by the fact that a similar minimum performance was found in all the analyses conducted in both experiments.

Taken together, the benefit in VWM performance seen in Experiment 1 seems to be derived from a two-step process in which grouping cues are processed pre-attentionally in the early stages of the visual stream, and impose a particular organization to the scene. This organization, automatically directs the attention (and attentional resources) to the grouped items due to an automatic feature-based attentional capture, leaving fewer resources for processing the rest of the information (Edward Awh & Jonides, 2001; Heuer & Schubö, 2016; Oberauer, 2002; Treisman, 1982; Vecera, 1994). This leads to an improvement for the grouped stimuli, with no performance boost or even a detrimental effect for non-grouped items.

In Experiment 2, the main objective was to explore whether the encoding bias found in Experiment 1 could be voluntarily counteracted by the current task goals. To this end, we performed the same CDT, but this time, the participants were instructed to ignore grouped items as they were irrelevant to the task. Accordingly, if the participants were able to filter out the grouped items we should find an opposite pattern of results compared to Experiment 1, with a better performance in those trials in which two grouped items were part of the memory array (Allon et al., 2019; Sawaki & Luck, 2010, 2011). This opposite pattern of results is what we found in Experiment 2. Particularly, the change detection performance increased in GA trials. However, a closer look at the results revealed that this benefit was only significant when no retrocue was presented. A likely explanation for this is that the presence of a cue in both short- and long-retrocue conditions helped NGA trials to maintain a similar performance in the CDT by signaling the target before it vanishes from VWM (Heuer & Schubö, 2016). On the other hand, when no retrocue was available GA trials outperformed NGA trials as participants were able to filter out the grouped items as irrelevant (see Fig. 3). Interestingly, when grouped items became irrelevant due to the task goals, the different retrocue conditions (short and long) were equally effective regardless of the presence of grouped elements in the array. This contrasts with Experiment 1, where long retrocues were less effective for NGP in GA (see Fig. 2 right). Taken together, the evidence from Experiment 2 supports the ability of the participants to voluntarily override the attentional capture caused by the presence of grouped items according to the task goals. This result is congruent with those found with other grouping principles in similar tasks (Allon et al., 2019), and contrasts with the effects found in time-based presentations (Zupan & Watson, 2020).

Sawaki and Luck (2010) proposed a model of how salient singletons (i.e., stimuli that contain unique feature values) capture attention in a stimulus-driven manner and the degree to which the top-down mechanism can attenuate or modulated this capture that is fully congruent with the results of the present study. In their model, the authors posit that salient items generate an attentional capture signal that, in the absence of top-down control, automatically attracts attention (the bottom-up saliency hypothesis). This automatic deployment can be avoided by a top-down active suppression process that is contingent on the imposed task goals (the contingent involuntary orienting hypothesis). This account can explain the results found in Experiment 1 (through an automatic attentional capture provoked by the salience of previously grouped elements) and the opposite pattern found in Experiment 2 (due to the active suppression imposed by the task instructions).

However, even though our results support the encoding bias hypothesis derived from the attentional capture generated by grouped items, we cannot rule out that the two mechanisms were working together at different stages of processing. It is feasible to consider the early organization of the visual scene as a “chunking” process in which: 1) items that share a common feature are treated as a single object, but 2) also lead to a feature-based attentional capture that biases the encoding of information into VWM.

Finally, although our study was not explicitly designed to investigate the limits of information storing and maintenance in VWM, the results of Experiment 1 are consistent with a flexible resources account of the working memory capacity and the role of attention in the VWM performance. In a recent study, Emrich et al. (2017) proposed a model of VWM limitations in which performance was determined by the proportion of attentional resources allocated to the items during encoding. This account offers a good explanation of both, the greater performance for GP and the differences in retrocue effectiveness between GP and NGP as retrocue latencies become larger. Specifically, the differences in change detection performance between grouped and non-grouped items increased with retrocue latency, indicating that both memory representations behave differently over time, a result that can be accounted from a flexible resources perspective, in which the quality and resolution of memory representations depend on the degree of attentional resources allocation (see also Bays & Husain, 2008; Brady et al., 2011, 2016). The results of Experiment 2 were also compatible with the attentional resources account, but given that grouped items were never probed, we are not able to discern whether these items received a lower amount of attentional resources or whether they were simply ignored and not encoded at all (which would lead to chance level performance). Conversely, from a discrete resources point of view, the storage of information is an all-or-none process, that either creates a representation at a fixed level of detail or no representation at all (Zhang & Luck, 2008). According to this model, once an item enters VWM, the level of precision and the strength of the memory trace should be the same for all the information stored, so we would expect to find similar differences between grouped and non-grouped probes in long- and no-retrocue trials.

## Conclusions

In the present study, we found evidence to support that the nature of the improvements in VWM when part of the information can be grouped through perceptual grouping cues, is not derived from optimized processing of the information to be remembered, but from an attentional encoding bias that greatly favors the encoding, maintenance and recovery of grouped items at the cost of worsening performance for the rest of the information. Also, we found that this attentional encoding bias can be overridden by top-down processes contingent on the current task goals. Overall, these two findings are congruent with the dual model of attentional capture by salient items proposed by Sawaki and Luck (2010), in which grouped items automatically generate an attend-to-me signal independent of task goals, but this signal can be overridden by top-down suppression processes that prevent the actual capture of attention and filter out the irrelevant information. In addition, our study indirectly supports a flexible-resources account of VWM storage based on the attentional resources allocated to the items, as shown by the interactions between the different latencies of retrocue presentations and the type of items cued.

## Data Availability

All data and materials are available on request.

## Code availability

The E-prime code created for the study is available on request.
